# Revision of the Laws of the Constitution

**Published:** 1913-06-28

**Authors:** 


### METROPOLITAN HOSPITAL SUNDAY FUND.

### Revision of the Laws of the Constitution.

### THE EFFECT OF THE AMENDMENTS.

A meeting of constituents of the Fund was held at the Mansion House on Friday, June 20, under the presidency of the Right Hon. the Lord Mayor, Sir D. Burnett, "to receive, and, if approved, to adopt the amended Laws of the Constitution of the Fund, drawn up in pursuance of the Council's resolution of January 29, 1913."

The Chairman of the General Purposes Committee, Prebendary Anderson, formally moved the adoption of the Laws of the Constitution as amended, remarking that they had received the careful attention of the Committee, and had been confirmed by the General Council.

The Rev. W. Hardy Harwood seconded, and pointed out the chief change effected by the amendments. Parts of the Constitution which were formerly scattered over two pages were now arranged under certain heads, and attempts had been made to render the constituency a little more real. Formerly, the meeting of constituents was practically a public meeting, at which anybody could walk in and vote. Moreover, no attempt was made to ensure that the representatives to that meeting had been chosen with care, and he feared that sometimes tickets were used without the particular will of the congregation. It was now asked that congregations would choose their representatives and supply their names to the Fund for record, the ordinary custom in the choosing being still perpetuated. Few realised how large the constituency was. If all who had a right to attend did so, Olympia or the Albert Hall would have to be hired, for they numbered 6,425. That number would be reduced, with the object of making the representation more real. No one wished to make money a basis for spiritual purposes, but this Fund was for ensuring the distribution of the money, and the only basis of graduation was the contribution of the congregations. Those which contributed under £5 numbered 982; over £30, 687; thus no one could say an undue representation was given to the larger contributors. Everyone would agree that the actual work of the Fund must be done by the Council. It was provided that one-fifth of the Council should retire each year, and their names should be submitted to each annual meeting before re-election, as in the case of a company meeting. The constituents would have the opportunity of challenging the action of the Council, either instructing that a certain action should not be repeated or rejecting the Council. For special meetings, which should naturally be very rare, it was thought that not less than 3 per cent., or 100 constituents, should have the power to call such. The effort had been to increase the harmony of working in a Fund where men differing very widely on important matters had one object in view —the relief of the sick poor of this great Metropolis.

Mr. Scott (Clapham) pointed out that under Regulation C all that the constituents had power to do was to pass the resolution for the election of the Council and the honorary officers; all the other resolutions must be in the form of recommendations to the Council.

The Rev. W. H. Harwood pointed out that what he spoke of had reference to a resolution which might be brought up suddenly in a meeting, but which should be considered by the Council, and brought back again to the constituents.

The Lord Mayor then went through the various clauses and invited comments on each as he reached them.

The Rev. L. S. Lewis (St. Mark, Whitechapel) asked if it was proposed to continue to hold the annual meeting in December.

Sir William Church pointed out that the amounts had been paid to the hospitals at the earliest possible date. If the constituents felt dissatisfied with any of the awards made it was open to them, at the annual meeting, to object to the proceedings of the Council, and to nominate other persons.

The Rev. L. S. Lewis said the Council could ignore the representations of the constituents or not as they pleased. Moreover, not more than one-fifth of the constitution of the Council could be altered in any one year. He moved that the annual general meeting be held within one week of the date on which the Council approved of the awards of the Distribution Committee, and before the awards were paid.

Mr. Counsell (Temple) seconded.

Sir William Church said he did not think all the constituents were aware of the machinery of the Fund. The proportionate grant to the hospitals was settled before the collections were made, and it was a mistake to suppose that all the money came in immediately after the collections were made. Owing to the kind services of ministers throughout the Metropolis, many people who were unable to give to the collections in churches subscribed largely to the Fund, and their subscriptions often were not sent in until considerably after Hospital Sunday. It was always found difficult to clear up the accounts of the Sunday collection until a good many weeks after the collection had been made in the churches.

The amendment was put and lost.

The Rev. L. S. Lewis objected to the requirement of 100 persons being necessary to call a special meeting. He proposed that the number be 20, and it was seconded. On being put it was lost.

The meeting accepted an amendment that where the duly accredited representative of a congregation could not attend, a properly elected substitute could be sent.

The Rev. L. S. Lewis then successfully pleaded for amendment of Sub-section (*c*), Law 6, relating to resolutions submitted to the Council, giving a subsequent meeting of constituents power to accept or reject resolutions submitted to them by the Council.

With regard to the election of the Council, after considerable debate Law 8 was somewhat radically altered.

A discussion also ensued on Law 15, which runs: "In estimating the amount to be contributed to the funds of any hospital from the Hospital Sunday Fund, no moneys paid or subscribed during the preceding year towards the school attached to any hospital shall be taken into account." The Lord Mayor pointed out that this was a short rule framed in such simple terms as to be readily understood by the public, but, if the meeting desired, the longer form could be used. The latter, taken from the report of Sir Edward Fry's Committee, ran: "For the future the distinction between the hospital and the school should in every case be drawn not only definitely and exactly, but with such clearness that it may be understood by the general public, and so that no question may arise as to the destination and application of moneys contributed, whether by the King's Fund or from any other source."

The Rev. L. S. Lewis said everyone would be delighted to learn the decision of the Council on that point, and it was to be hoped that there would be no mistake. He said so because people of ability had taken Law 15 to mean the opposite of that which it was now said to mean; in fact, out of six people only one thought it bore the meaning which had just been placed upon it. He suggested the following words: "If any sum of money has been paid from the Hospital General Fund to the school attached to it during the preceding year otherwise than for laboratory work in connection with sick patients, which would have been paid for elsewhere, the grant shall be withheld until such time as that school shall have repaid that sum to the hospital." He had no desire to rake up an old controversy, but reminded the meeting that when the King's Fund withheld its grant from St. George's Hospital the Sunday Fund greatly increased its grant to that hospital. There should be no ambiguity about the wording now the Laws were being put right.

Canon Pearce contended that this rule as now submitted by the Council would put the Sunday Fund into line with the King's Fund; there was an immense advantage in uniformity. And he believed Mr. Lewis's proposed amendment to be a much weaker law than the Council's.

The Rev. L. S. Lewis wished to put a specific question to Sir William Church: Supposing a hospital spent £10,000 on its hospital in any one year, and £1,000 on its medical school, and the grant were 10 per cent. of receipts of the Fund for that institution, would the grant be 10 per cent. on £10,000 or on £11,000?

Sir William Church replied that if the £1,000 supposed appeared in the accounts, they would not be taken into consideration by the Fund. But hospitals could have a discretionary fund over which they had free control. But the Sunday Fund did not go so closely into hospital management as did the King's Fund. In the case of St. George's Hospital, what the King's Fund found was that certain work done was being paid for too highly.

Sir A. Pearce Gould said the obvious short answer to Mr. Lewis's question was that the 10 per cent. grant would be on the £10,000.

The amendment was put and lost.

The whole revised Laws were then put and carried.

On the motion of the Archdeacon of London, the Lord Mayor was heartily thanked for his patient and tactful conduct of the meeting.

### THE REVISED LAWS OF THE CONSTITUTION.

### A. Constituents.

1. The Constituents of the Metropolitan Hospital Sunday Fund shall be the duly elected representatives of the Congregations which contribute annually to the Fund.

2. These Congregations which have contributed to the Fund during two successive years shall be entitled to a voice in its management during the year next following the last of such contributions, on the following basis of representation:—

(*a*) Congregations contributing Five Pounds and under to be represented by the Clergyman, Minister, or duly appointed representative of such Congregation, chosen in accordance with the usual custom of such Congregation.

(*b*) Congregations contributing from Five Pounds to Thirty Pounds to be represented by the Clergyman or Minister of such Congregation, and one Lay representative chosen as aforesaid.

(*c*) Congregations contributing over Thirty Pounds to be represented by the Clergyman or Minister of such Congregation, and two Lay representatives appointed as aforesaid.

(*d*) The Council may invite any donor of not less than £20 to the Annual General Meeting of the Fund, and such donors shall have the power to vote at such Meeting.

### B. Meetings of Constituents.

3. A General Meeting of the duly elected representatives of contributing Congregations shall be summoned to meet the Council each year in the month of December at such hour as the Council may determine to receive and adopt the Annual Report of the Council, and to fill up the vacancies on the Council for the succeeding twelve months.

4. The Council may, when it appears to them desirable, and shall on the written requisition of not less than 100 duly appointed representatives of the contributing Congregations call a Special General Meeting of such representatives. Notice of such General Meetings shall be sent by post to the last known address of the Clergyman, Minister, or representative at least Fourteen days before the day of Meeting. And every such notice shall specify the objects for which such Meeting is called.

5. As soon as possible after the date fixed for Hospital Sunday in each year, and in any case not later than June 30, the Clergyman or Minister of each contributing Congregation or his duly appointed substitute shall send to the Secretary of the Fund the name and address of the representative or representatives duly appointed by his Congregation for the current twelve months.

6. At each Meeting of the Constituents the Chairman of the day shall notify:—

(*a*) That persons desirous of taking part in the discussion are requested to send up their names to the Chairman.

(*b*) That speeches will be limited to Ten Minutes.

(*c*) That after the passing of the resolutions proposing the election of the Council and Honorary Officers, and the adoption or rejection of the Report, and the usual complimentary votes, all other resolutions must be in the form of recommendations to the Council, by whom all such recommendations shall be carefully considered, and the result of such consideration be reported to a subsequent Meeting of the Constituents, for acceptance or rejection.

### C. The Council.

7. The Council shall consist of not more than Fifty Clerical and Fifty Lay Members in addition to the President (who shall be the Lord Mayor for the time being), Vice-President, the Members of the Committee of Distribution, and the Hon. Secretaries, who shall *ex-officio* be Members of the Council.

8. One-fifth of the Members of the Council shall retire annually in rotation, but shall be eligible for re-election, the order of rotation in the first instance to be determined by Ballot of the Council. The Notice convening the Annual Meeting of Constituents shall contain the names of those Members who are eligible and recommended for re-election, and any new names suggested, such names to be sent in on or before November 30.

9. The Vice-Chairman, Members of the Committee of Distribution, and of all other Committees, shall be elected each year at the first Meeting of the Council.

10. The Council shall have power to make all arrangements for the collection on Hospital Sunday, to appoint all Committees, and to frame such By-laws as may be needful for the proper administration of the Fund.

### D. Committee of Distribution.

11. The Committee of Distribution shall consist of the Lord Mayor as President, the Hon. Secretaries *ex-officio*, and of Nine other Members.

12. The Committee of Distribution shall present their Report to the Council for approval before the Fund is distributed, and when so approved the awards shall be forthwith paid. A copy of the Report shall be sent to each Member of the Council at least seven days before its formal reception.

13. The Committee of Distribution shall have power to make awards, not exceeding in the aggregate five per cent. of the total sum at the disposal of the Committee in any year, to District Nursing Associations, employing fully-trained Hospital Nurses, and to hold in reserve for the purpose of grants towards the purchase of Surgical Appliances for the Poor of the contributing Congregations an amount not exceeding seven and a half per cent. of the said total sum.

14. The amount of any collections made at Places of Worship on Hospital Sunday and forwarded to any participating Institution direct shall be deducted from the award to such Institution. And should any such Institution press its claims upon the Public during the fortnight preceding Hospital Sunday, the Committee of Distribution shall have power to make such deduction from its award as they in their discretion may think fit.

15. In estimating the amount to be contributed to the funds of any Hospital from the Hospital Sunday Fund, no moneys paid or subscribed during the preceding year towards the School attached to any Hospital shall be taken into account.

### E. Participating Institutions.

16. Those Hospitals and Dispensaries only which are managed by a Committee duly constituted, and which produce their printed Reports with Income and Expenditure Accounts and Balance Sheets duly audited for the last Three Years by a Public Accountant, in accordance with the Uniform System of Accounts agreed upon, shall be allowed to participate in the Fund, and no Institution, the benefits of which can only be gained by election of the subscribers, shall so participate.

### F. Methods of Distribution.

17. The awards to Institutions shall be primarily based upon the average ordinary expenditure thereof for the preceding Three Years, after deducting therefrom the amount of the income from estates, endowments, investments, legacies exceeding £100 not necessarily absorbed in current expenditure, and the cost of administration. But in every case the Merits and pecuniary Needs of each Institution shall be fully inquired into by the Distribution Committee, and the basis of award shall be determined by them upon such Needs and Merits. No basis of award shall be subject to any reduction beyond those above provided without notice to the Institution affected, or until a conference between the Managing Body of such Institution and the Distribution Committee.

In the case of Hospitals having an efficient Inquiry Officer to detect abuse in the Out-Patient Department the same shall be regarded as an important factor in determining "Merits."

18. The payments made by or on behalf of Patients shall be dealt with as the Committee of Distribution may determine.

